# Use of connectotyping on task functional MRI data reveals dynamic network level cross talking during task performance

**DOI:** 10.3389/fnins.2022.951907

**Published:** 2022-10-10

**Authors:** Valeria Vazquez-Trejo, Binyam Nardos, Bradley L. Schlaggar, Damien A. Fair, Oscar Miranda-Dominguez

**Affiliations:** ^1^Rush Medical College, Chicago, IL, United States; ^2^Department of Biology, Portland State University, Portland, OR, United States; ^3^Department of Behavioral Neuroscience, Oregon Health and Science University, Portland, OR, United States; ^4^Department of Occupational Therapy, Washington University School of Medicine in St. Louis, St. Louis, MO, United States; ^5^Kennedy Krieger Institute, Baltimore, MD, United States; ^6^Department of Neurology, Johns Hopkins University School of Medicine, Baltimore, MD, United States; ^7^Department of Pediatrics, Johns Hopkins University School of Medicine, Baltimore, MD, United States; ^8^Department of Pediatrics, Medical School, University of Minnesota, Minneapolis, MN, United States; ^9^Masonic Institute for the Developing Brain, University of Minnesota, Minneapolis, MN, United States; ^10^Institute of Child Development, College of Education and Human Development, University of Minnesota, Minneapolis, MN, United States; ^11^Minnesota Supercomputing Institute, University of Minnesota, Minneapolis, MN, United States

**Keywords:** fMRI, task fMRI, connectotyping, functional connectivity, cognition, dynamic connectivity, widely spaced event-related fMRI, BOLD

## Abstract

Task-based functional MRI (fMRI) has greatly improved understanding of brain functioning, enabling the identification of brain areas associated with specific cognitive operations. Traditional analyses are limited to associating activation patterns in particular regions with specific cognitive operation, largely ignoring regional cross-talk or dynamic connectivity, which we propose is crucial for characterization of brain function in the context of task fMRI. We use connectotyping, which efficiently models functional brain connectivity to reveal the progression of temporal brain connectivity patterns in task fMRI. Connectotyping was employed on data from twenty-four participants (12 male, mean age 24.8 years, 2.57 std. dev) who performed a widely spaced event-related fMRI word vs. pseudoword decision task, where stimuli were presented every 20 s. After filtering for movement, we ended up with 15 participants that completed each trial and had enough usable data for our analyses. Connectivity matrices were calculated per participant across time for each stimuli type. A Repeated Measures ANOVA applied on the connectotypes was used to characterize differences across time for words and pseudowords. Our group level analyses found significantly different dynamic connectivity patterns during word vs. pseudoword processing between the Fronto-Parietal and Cingulo-Parietal Systems, areas involved in cognitive task control, memory retrieval, and semantic processing. Our findings support the presence of dynamic changes in functional connectivity during task execution and that such changes can be characterized using connectotyping but not with traditional Pearson’s correlations.

## Introduction

Task-based functional MRI (fMRI) has had a profound impact on our understanding of brain functioning. Using fMRI, it is possible to design experiments that target specific sensorimotor, perceptual, and/or cognitive operations in efforts to understand the brain’s basis of those functions. Complementing neuroscientific findings based on other methods (e.g., single cell or multiunit recording), and lesion cases, task-based fMRI studies have identified functional neuroanatomy underlying various sensorimotor and perceptual systems. Examples include visual (Engel et al., 1994; Sereno et al., 1995; Goebel et al., 1998) and auditory systems (Moerel et al., 2014), as well as systems associated with higher-order cognitive operations such as memory retrieval (Yonelinas and Levy, 2002; Wheeler and Buckner, 2003; Dobbins and Wagner, 2005; Yonelinas et al., 2005; Cabeza et al., 2008; Nelson et al., 2010; Rugg and Vilberg, 2013), semantic processing (Petersen et al., 1988; Fiez, 1997; Thompson-Schill et al., 1997; Friederici et al., 2000; Donaldson et al., 2001; Roskies et al., 2001; Wagner et al., 2001; Badre et al., 2005; Gordon et al., 2014), and cognitive control (Botvinick et al., 2001; Braver and Barch, 2006; Dosenbach et al., 2006, 2007).

The primary measure in fMRI studies is the blood oxygen level dependent (BOLD) signal. Although not a direct measure of neural activity, it has been shown that the measured BOLD signal is correlated with neural activity, particularly with local field potentials (Logothetis et al., 2001; Logothetis, 2003). The BOLD signal, however, is slow compared to neural activity. After an initial stimulus, the BOLD signal peaks typically after 6 s (Vazquez and Noll, 1998), returning to baseline in approximately 20 s –this observed activation trend is known as the hemodynamic response function. The signal delay in returning to baseline needs to be considered in experimental design (Logothetis, 2008). For example, in a typical task experiment, participants are exposed to a given stimulus (cognitive, visual, or auditory) or are asked to perform a task. Given the knowledge of the delayed peak on activation, methods are tuned to look for brain areas that respond specifically to the experimental paradigm once peak response is achieved.

The subsequent development of resting state functional connectivity MRI (rs-fcMRI) was another milestone in neuroimaging. Biswal et al.’s seminal work (Biswal et al., 1995) established that the low frequency (<0.1 Hz) resting BOLD activity in brain regions that are typically coactivated during task-states (or known to be members of a common brain system e.g., left and right primary motor cortex) show a high degree of temporal correlation. This high degree of correlation is hypothesized to be a measure of functional connectivity among the said regions. rs-fcMRI has since become a very convenient technique to characterize brain function. Since it does not require the presence of an overt cognitive task, it can be employed in animals (Miranda-Dominguez et al., 2014b; Stafford et al., 2014), developmental populations (Marek et al., 2019), or in patients that may otherwise be unable to perform intentional cognitive tasks.

There is a growing interest in characterizing dynamic changes in brain connectivity (Chang and Glover, 2010), both at rest and during tasks. Several groups have used different techniques to characterize the cross-talking between brain areas (Friston et al., 1997; Chang and Glover, 2010; Ginestet and Simmons, 2011; Cribben et al., 2012; Lindquist et al., 2014; Billings et al., 2021; Shappell et al., 2021) but there are controversies in the field (Laumann et al., 2016). One of the first methods to estimate dynamics in functional connectivity is the use of “sliding windows” (Sakoğlu et al., 2010), where the BOLD data is split in segments, connectivity is calculated on each segment, then changes in functional connectivity are tracked across time. This has been mostly used in resting state data. Another approach is to assume that functional connectivity is a dynamic process that can be characterized by a multivariate gaussian distribution whose mean and covariance matrix evolves on time and there are efficient algorithms that can estimate those statistical properties (Xu and Lindquist, 2015). Dynamic Causal Modeling (DCM), (Friston et al., 2003, 2019), is another technique that models dynamic changes in functional connectivity. DCM can be applied on resting state and task data. In DCM, the user specifies the brain areas that define “the circuit” involved in a task and then, by using differential equations and non-linear dynamics, a predicted hemodynamic response is modeled and compared against the measured signal within the proposed circuit. An alternative method is to use non-linear dynamics to identify transitions among different states in timeseries (Zhang and Saggar, 2020). There have also been attempts to characterize dynamic changes in connectivity in task-fMRI experiments. An approach that has been used by some but not all studies, relies on averaging data across participants to increase signal to noise ratio (intrasubject correlation analysis). In this method, dynamic changes in connectivity are estimated after calculating the correlation of each brain area’s timeseries against each other across participants and averaging correlations across participants (Hasson et al., 2004; Najafi et al., 2017). There are also methods such as Psychophysiological Interactions (PPI, McLaren et al., 2012) and Rissman connectivity (Rissman et al., 2004) that do not rely on averaging data across participants but need a brain area or areas as seed(s) to estimate changes in connectivity in reference to that seed. The most basic implementation of PPI (McLaren et al., 2012) consists of defining 3 regressors, the timeseries of a) the task, b) the BOLD response of the seed, and c) the product of those 2 signals. The BOLD response of brain areas that can be modeled by the product of the 2 signals considered to be functionally connected to the seed and be involved in the task. The other 2 regressors are used to control for areas that respond to the task but are not connected to the seed and for areas that are in close proximity to the seed but not functionally involved in the task. In Rissman connectivity, the BOLD data is aligned according to the timing of events and, using the Generalized Linear Model framework, beta-weights associated with those events are calculated for every voxel (Rissman et al., 2004). Resulting event-related beta-weights are correlated across voxels to identify connections associated with the task being studied.

One may reasonably hypothesize that there are dynamic functional connectivity changes on the networks supporting any mental process that may occur on the order of seconds during the instantiation, computation, and response frame of a given task. Hence, there is a need for a method able to utilize whole brain connectivity to identify the brain networks that support a task. This could be done by aligning the BOLD data according to the phase of a task, calculating instantaneous connectivity at each phase and tracking changes on time across networks. Unfortunately, one of the main problems in fMRI is that the BOLD signal is highly susceptible to noise and correlations. The traditional method used to characterize whole brain connectivity, may not have the resolving power to unveil dynamic changes in connectivity.

**FIGURE 1:**
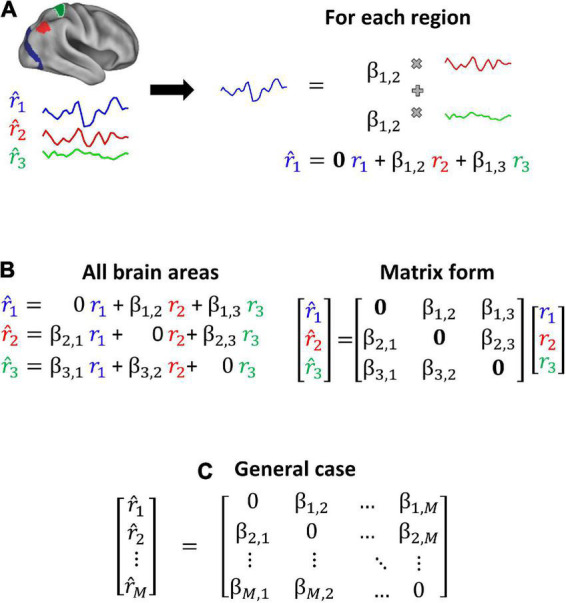
Connectotyping. Functional connectivity is calculated as the weighted sum of activity from the remaining brain regions (Miranda-Dominguez et al., 2014a). **(A)** Given 3 brain areas, each timeseries is modeled as the weighted sum of the other timeseries. **(B)** All the equations can be expressed as a matrix and solved using linear algebra. **(C)** This approach can be generalized to *M* brain regions.

Connectotyping (Miranda-Dominguez et al., 2014a,^2018^), a model-based method used to calculate functional connectivity has the potential to address the above limitations. We have shown that connectotyping is able to identify personalized patterns of brain connectivity with an improved signal-to-noise ratio even when using limited amounts of data as demonstrated in a recent study where this approach was used to characterize heritable patterns of brain functional connectivity (Miranda-Dominguez et al., 2018). Connectotyping is based on a linear model that proposes that the activity of a given brain region can be described by the weighted sum of all the other brain regions (Figure 1). The coefficients (beta-weights) of the resulting model correspond to a connectivity matrix that is capable of identifying a functional fingerprint in individual participants using a small amount of data (e.g., 5 min of rs-fcMRI), which is the typical amount of movement-free data able to be acquired in most studies.

The aim of this current study is to determine whether connectotyping can be applied to a task fMRI dataset to track changes in network-network functional connectivity *during the progression of a task*. This study relies on the following three assumptions: (1) A given task will activate specific brain areas and networks tuned to respond to the different aspects of the progression of such task. (2) As the task evolves, the balance –or co-activation patterns— among brain areas will change, reflecting dynamical tuning to different aspects of the task. (3) There is a contrast in the task that enables the differentiation of pure activation of brain areas versus task-specific changes in brain connectivity. With connectotyping, the activity of each brain area is modeled as the weighted contribution of all the other brain areas. Therefore, we can use connectotyping to capture instantaneous connectivity maps as the task evolves. Then, dynamic changes in functional connectivity secondary to the evolution of the task can be characterized using statistical testing (repeated measures ANOVA tests).

For this analysis, we first identified a task-fMRI dataset with stimuli presented in a widely spaced manner without the confounding effect of BOLD activity overlap from past stimuli. Again, one of the characteristics of the BOLD signal that must be considered in this context is that it takes about 20 s for the hemodynamic response function to return to baseline following stimulus presentation. However, we avoid overlapping responses by using data from widely spaced event-related fMRI experiment (at least 20 s between individual stimuli) in which subjects were performing a visually presented word vs. pronounceable non-word (hereafter pseudoword or PW) lexical decision task (Nardos, 2015).

Specifically, we hypothesized that connections between networks implicated in cognitive control (Botvinick et al., 2001; Braver and Barch, 2006; Dosenbach et al., 2006, 2007), memory retrieval (Iidaka et al., 2006), and semantic processing (Petersen et al., 1988; Fiez, 1997; Thompson-Schill et al., 1997; Friederici et al., 2000; Donaldson et al., 2001; Roskies et al., 2001; Wagner et al., 2001; Badre et al., 2005) would have dynamic network-network functional connectivity differences as a function of the type of stimulus (word vs. PW) being processed.

In summary, our goal is to track changes in functional connectivity between different functional networks. We hypothesized that the distinction between word and PW relies on the dynamic activation of higher order attention networks and that connectotyping has enough resolving power to characterize such changes and can do so better than using connectivity matrices created *via* Pearson’s correlations.

## Materials and methods

### Participants

The original study sample consisted of 28 participants; after excluding participants who had incomplete or compromised data quality, the current study included 24 individuals. Participants were 24 monolingual (English-speaking), right-handed participants (12 male, mean age 24.8 years, 2.57 std. dev) recruited from neighborhoods surrounding Washington University in St. Louis as well as from the university student body (Nardos, 2015). All participants had no history of psychiatric or neurological illness and scored above the 50th percentile on the Woodcock-Johnson III reading assessment (Woodcock and Johnson, 2002). The Washington University Human Studies Committee approved the study (IRB ID # 201202083) and all participants were reimbursed for their participation.

### Task

In a visually presented lexical decision task, individuals identified words vs. PWs while in the MRI scanner *via* button pressing. A set of words (50% animals; 50% artifacts; 3–9 letters; 1–3 syllables) and PWs (5 letters, 1 or 2 syllables) were selected from the English Lexicon Project (Balota et al., 2007; Nardos, 2015). Pseudowords were vetted by an expert, ensuring that words and pseudowords were tightly matched on lexical characteristics like number of letters, number of syllables, bigram frequency, and orthographic neighborhood size (Nardos, 2015). When in the scanner participants had two buttons, one on each hand. Each button corresponded either to words or PWs, participants pressed the buttons with the thumb of either hand to identify the stimuli. Stimuli were presented in a widely spaced manner, i.e., separated by ∼20 s, to avoid hemodynamic response signal overlap across individual stimuli and allow extraction of individual trial BOLD responses (Nardos, 2015). In each trial, a word or PW stimulus was presented for 2.5 s (1 TR or MR frame) with each letter subtending 0.5° of horizontal visual angle, followed by 17.5 s (7TRs or MR frames) of a black fixation screen with a white cross. Participants underwent 10 functional MRI runs each with 24 stimuli (18 PWs and 6 words) per run. Out of 24 trials within a run, 3 of those trials were catch trials, meaning that the intertrial interval after those trials would randomly be 2, 3, or 4 times the duration of the TR (2.5 sec), i.e., 5, 7.5, or 10 s, respectively. Catch trials were run for both words and pseudowords. While there were differences across participants in reaction time, overall accuracy was very high (98%, see (Nardos, 2015) for details).

### Data acquisition

Structural and functional MRI data were collected as described in Nardos (2015) from a Siemens 3 Tesla MAGNETOM Trio system (Erlangen, Germany). The scanner included total imaging matrix technology (TIM) and utilized a 12-channel head matrix coil. A high resolution T1-weighted MP-RAGE was acquired (TE = 3.08 ms, TR [partition] = 2.4 s, TI = 1,000 ms, flip angle = 8′′, 176 slices with 1 × 1 × 1 mm voxels). To improve atlas alignment a T2-weighted turbo spin echo structural image (TE = 84 ms, TR = 6.8 s, 32 slices with 2 × 1 × 4 mm voxels) matching the acquisition plane of the BOLD images were also collected. Alignment to the anterior commissure-posterior commissure (AC-PC) plane was performed by Siemens pulse sequence protocol. BOLD contrast-sensitive gradient echo echo-planar sequence (TE = 27 ms, flip angle = 90′′, in-plane resolution = 4 × 4 mm) was used for functional data collection. Using a TR of 2.5 s, 32 contiguous, 4 mm- thick axial slices whole-brain EPI volumes were collected. Communication with participants was facilitated by MR- compatible headphones which were also used to reduce noise from the scanner. Head movement was minimized by using a molded thermoplastic mask. Stimuli were presented using Psyscope (Cohen et al., 1993) installed on an iMAC computer (Apple, Cupertino, CA) and projected *via* an LCD projector (Sharp model PG-C20XU) onto an MRI-compatible rear-projection screen combined with a mirror attached to the head coil (CinePlex).

### MRI data preprocessing

Data were processed using surface-based registration applying a modified version from the Human Connectome Project pipeline (Glasser et al., 2013) plus in-house denoising methods.^1^ Processing includes the use of FSL (Smith et al., 2004; Woolrich et al., 2009; Jenkinson et al., 2012) and FreeSurfer tools (Sereno et al., 1995; Fischl and Dale, 2000; Desikan et al., 2006). Briefly, gradient distortion corrected T1-weighted and T2-weighted volumes were first aligned to the MNI’s AC-PC axis and then non-linearly normalized to the MNI atlas. Later, the T1w and T2w volumes were re-registered using boundary-based registration (Greve and Fischl, 2009) to improve alignment. Individual brains were segmented using recon-all from FreeSurfer. Segmentations were improved by using the enhanced white matter-pial surface contrast of the T2-weighted sequence. Additionally, the initial pial and white matter surfaces were used to distinguish an initial cortical ribbon. From these segmentations, a tailored 3D surface was created for each participant and registered to the Conte 69 surface atlas of the Human Connectome Project.

The BOLD data were corrected for field distortions (using FSL’s TOPUP) and processed by doing a preliminary 6 degrees of freedom linear registration to the first frame. After this initial alignment, the average frame was calculated and used as a final reference. Next, the BOLD data were registered to this final reference and to the T1-weighted volume, all in one single step, by concatenating all the individual registrations into a single registration. To allow steady state magnetization, the first four volumes of each run were discarded. The cortical ribbon defined by the structural T1-weighted and T2-weighted volumes was used to define a high-resolution mesh used for surface registration of the BOLD data. This cortical ribbon was also used to quantify the partial contribution of each voxel in the BOLD data in surface registration. Timecourses in the cortical mesh were calculated by obtaining the weighted average of the voxels neighboring each vertex within the grid, where the weights are given by the average number of voxels wholly or partially within the cortical ribbon. Voxels with a high coefficient of variation, indicating difficulty with tissue assignment or containing large blood vessels, were excluded. Next, the resulting timecourses in this mesh were downsampled into a standard space of 91, 282 anchor points (grayordinates), which were defined in the brain atlas and mapped uniquely to each participant’s brain after smoothing them with a 2 mm full-width-half-max Gaussian filter. Subcortical regions were treated and registered as volumes. Two-thirds of the grayordinates are vertices located in the cortical ribbon while the remaining grayordinates are subcortical voxels. Subsequently, resulting timecourses (surface registration for cortex and volume registration for subcortical gray matter) were detrended. The following steps involved regression of (1) 6 degrees of freedom obtained by rigid-body head motion correction, (2) whole brain signal, (3) ventricular signal averaged from ventricular regions of interest (ROIs), (4) white matter signal averaged from white matter ROIs, (5) first-order derivative terms and the squares for whole brain, ventricular and white matter signals to account for variance between regressors. Finally, timecourses were filtered using a first-order Butterworth band-pass filter with frequency range from 9 to 80 mHz. This filter was applied in the forward and backward direction to remove phase distortions.

### Regions of interest and functional networks

**FIGURE 2:**
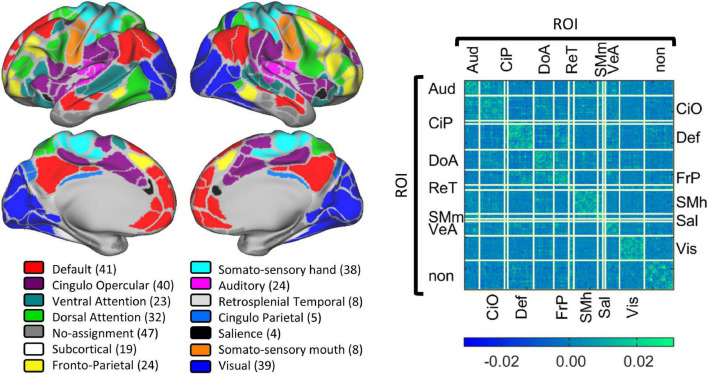
Gordon parcelation schema. The **(Left panel)** displays a visual representation of the 13 defined cortical networks in the Gordon Parcelation. Networks are color coded with the number of regions of interest shown in parentheses. The **(Right panel)** shows the average connectivity matrix calculated as the average beta-weights of all the data included in this study. Each row and column correspond to a particular brain region, and each cell is a connection. Connections are grouped by functional network pair.

Collected BOLD data were parcellated using the Gordon schema that has 333 regions of interest (ROIs) grouped into 12 networks (Gordon et al., 2014). Each grayordinate was assigned to a region and network within this parcelation. The networks, their abbreviation and the number of ROIs included are: Auditory (Aud, *n* = 24), Cingulo-Opercular (CiO, *n* = 40), Cingulo-Parietal (CiP, *n* = 5), Default (Def, *n* = 41), Dorsal Attention (DoA, *n* = 32), Fronto-Parietal (FrP, *n* = 24), Retrosplenial Temporal (ReT, *n* = 8), Somato-sensory hand (Sml, *n* = 38), Somato-sensory mouth (SMm, *n* = 8), Salience (Sal, *n* = 4), Ventral Attention (VeA, *n* = 23), and Visual (Vis, *n* = 39). From the 333 ROIs, 47 ROIs were not assigned to any network. The location of each functional network is shown in Figure 2. Per our hypothesis, on this pilot study we excluded primary somatosensory and unimodal networks and included only the Cingulo-Parietal, Default, Dorsal Attention, Fronto-Parietal, Salience, and Ventral Attention, ending up with 129 brain areas from 6 brain networks that were grouped into 36 functional network pairs (CiP-CiP, CiP-Def,…). Table 1 shows all the functional network pairs including the count of unique connectotyping’s beta-weights.

**TABLE 1:** List of all 36 functional network pairs tested in the ANOVA with the number of ROI connections between networks listed in the third column. Grouping of connections per functional system pair.

#	Name	Number of connections
1	CiP and CiP	20
2	Def and CiP	205
3	DoA and CiP	160
4	FrP and CiP	120
5	Sal and CiP	20
6	VeA and CiP	115
7	CiP and Def	205
8	Def and Def	1640
9	DoA and Def	1312
10	FrP and Def	984
11	Sal and Def	164
12	VeA and Def	943
13	CiP and DoA	160
14	Def and DoA	1312
15	DoA and DoA	992
16	FrP and DoA	768
17	Sal and DoA	128
18	VeA and DoA	736
19	CiP and FrP	120
20	Def and FrP	984
21	DoA and FrP	768
22	FrP and FrP	552
23	Sal and FrP	96
24	VeA and FrP	552
25	CiP and Sal	20
26	Def and Sal	164
27	DoA and Sal	128
28	FrP and Sal	96
29	Sal and Sal	12
30	VeA and Sal	92
31	CiP and VeA	115
32	Def and VeA	943
33	DoA and VeA	736
34	FrP and VeA	552
35	Sal and VeA	92
36	VeA and VeA	506

	Total	16,512

### Motion censoring

Correction for head motion was completed by calculating six parameters of head movement, movement and rotation along the x, y, and z axes. The absolute sum of movement along these parameters was evaluated after each change of frame and termed “frame displacement” (FD). For our study, we set our FD threshold at 0.3 mm and set the FD of the first frame at zero. This measure was only used as a way to detect motion and was not used for regression (Power et al., 2012; Siegel et al., 2014).

### Grouping data for connectotyping

**FIGURE 3:**
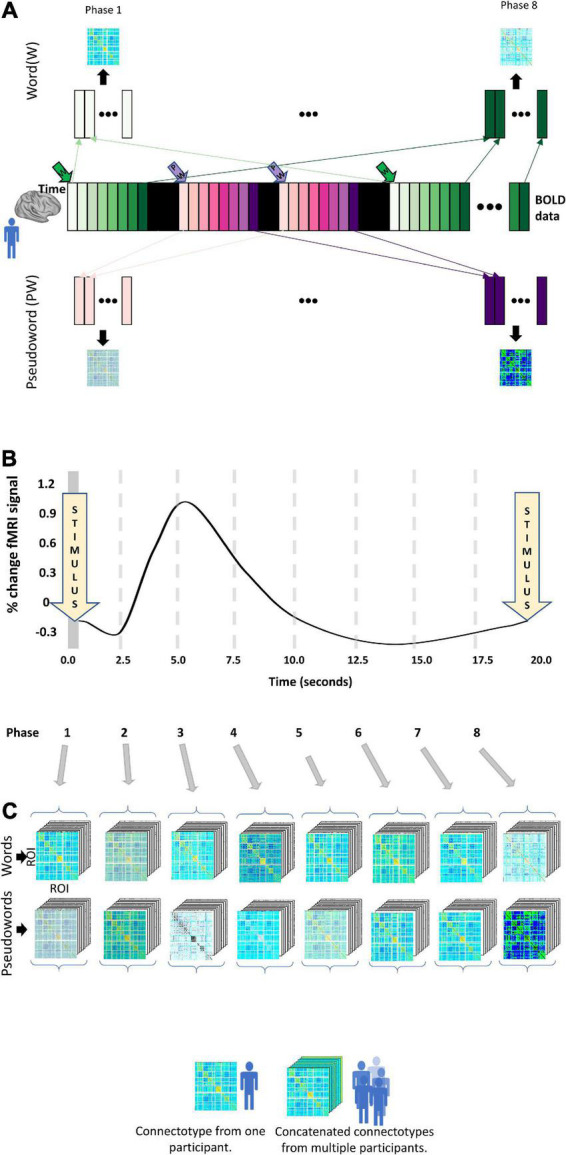
Experimental design. The widely spaced design of the word versus pseudoword experiment allows us to characterize dynamic changes in functional connectivity using connectotyping. **(A)** The collected whole-brain BOLD data is represented as the central colored strip. Each bar corresponds to data acquired at each TR and is color-coded according to the stimuli (Green for “Word” and purple for “PW”). A word or pseudoword is displayed for 2.5 s with no new stimuli after 20 s. The bars in black represent “catch” trials. Frames collected at the same time, relative to the exposure to the stimuli, are concatenated to create a stack of frames of size 333 times replica frames. Those frames are used to calculate a connectivity matrix. This approach generates 16 connectivity matrices per participant. **(B)** Temporal evolution of the theoretical hemodynamic response. **(C)** Resulting connectotypes from each participant **(A)** are grouped according to their phase and stacked with the connectotypes of all the participants included in the study.

We calculated instantaneous connectotypes for each participant at each phase (Frame 1–8) of the progression of each task (i.e., for words and PWs) ending up with 16 connectotypes per participant, as shown in Figure 3. Each resulting connectotype captures the instantaneous cross-talking among brain areas at each phase of the progression of each task. To do this, for each participant, at each frame and stimulus type, we concatenated the BOLD data from the same frame, relative to the frame at which stimuli was presented for each participant (as shown in Figure 3A). We did this because these replica frames correspond to the same point in time in the dynamic evolution of the task. This created a matrix with the dimension 333 by the number of replica frames. The dimension 333 is due to the number or ROIs included in the Gordon parcelation schema (Gordon et al., 2014). Next, we used that stack of replica frames to calculate a connectotype that reflects the crosstalking between ROIs at this phase of the task. This approach was repeated for each phase of the experiment. We only included trials that had a length of 8 TRs for a total of 20 s, with the additional constraint that the preceding trial in the experiment took place at least 20 s prior, ensuring that the timecourse for the current trial under consideration is not adulterated by that previous trial. Frames were excluded if head movement was higher than a given frame displacement (FD) threshold of 0.3 mm (Power et al., 2014). Participants were included only if they had enough data (40 replica frames or more) to calculate personalized connectotypes on each of the 16 conditions. Fifteen participants met this condition. Each connectotype was calculated using 40 “replica” frames to avoid the confounding factor that some connectotypes from specific participants could be calculated with different numbers of frames. For each condition and participant, we calculated connectotypes using 40 frames selected randomly within condition from the surviving frames with head movement lower than the pre-selected threshold. We decided to select frames randomly instead of the ones with the lowest FD to avoid bias and batch effects secondary to head movement. We ended up with 15 participants that successfully completed each trial (word and PW) for 20 s and had at least 40 low head-movement replica frames for each condition (16).

### Connectotyping

As described in the original publication (Miranda-Dominguez et al., 2014a), connectotyping mathematically represents a brain region’s signal as the weighted sum of the signal from every other brain region using values termed beta-weights (β). Such weights are optimized by regularization and cross-validation. The result is a directional connectivity map that calculates the interaction between brain regions, allowing the identification of individual connectivity patterns among brain areas and networks. Briefly, in an example application on a hypothetical parcelation schema with only three brain regions, “a,” “b,” and “c”; this technique models the functional connectivity of region “a” as a weighted sum of regions “b” and “c’s” connectivity. The model for the signal resulting from region “a” would be: $\hat{a}=\beta_{a,b}\,b+\beta_{a,c}\,c$. This same model is then applied to the remaining brain regions “b” and “c” until the signal for each region in the system is represented by an equation (see a schematic representation of connectotyping in Figure 1).

After applying the connectotyping approach to our filtered and grouped and timely aligned task-fMRI data, we ended up with 15 participants each with 16 connectotypes that characterize the instantaneous connectivity map as each trial evolved (word and PW).

To note, in the original manuscript (Miranda-Dominguez et al., 2014a), which aimed to characterize functional connectivity in resting state data, the first step was to account for the spurious effect of autocorrelations. In contrast, in task-based fMRI, autocorrelations are not spurious; they are part of the temporal evolution of the task. For this reason, in this study we did not remove autocorrelations from the data.

Additionally, while connectotyping created individualized connectivity patterns, our study performed analyses on concatenated connectotypes from 15 participants resulting in a group level analysis.

### Statistical analysis

**FIGURE 4:**
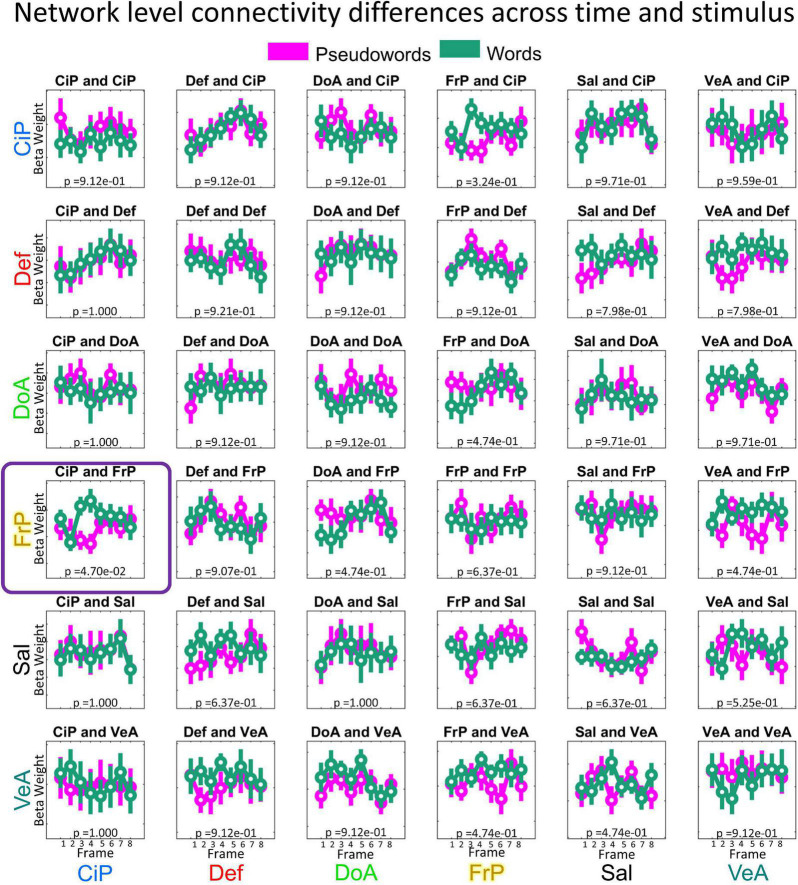
Distribution of connectivity values per functional network pair and condition. Beta-weights were calculated for each condition as indicated in our experimental design (Figure 3) and grouped by functional network pair. Each boxplot highlights the mean values using a circle and the dispersion is indicated with a bar covering 1.15 times the standard deviation of the connectivity values. Data is color-coded by stimuli: pseudoword (purple) and word (green). *X*-axis indicates the time, in frames (TR of 2.5 s each). In this study we included the following six networks: Cingulo-Parietal (CiP, *n* = 5 Regions of Interest), Default (Def, *n* = 41), Dorsal Attention (DoA, *n* = 32), Fronto-Parietal (FrP, *n* = 24), Salience (Sal, *n* = 4), and Ventral Attention (VeA, *n* = 23).

To identify dynamic changes in functional connectivity, we ran independent repeated measures ANOVA tests for each functional network pair (*N* = 36, as described before) testing for changes in functional connectivity for the interaction of time (frame 1 to 8) and stimulus type, (i.e., word/PW) using the 16 connectotypes from all the surviving participants (Figure 3). All statistical analyses were performed in MATLAB. Before statistical testing, connectivity values (i.e., connectotyping beta-weights) were box-cox transformed to normalize distributions (Montgomery, 2005) and the logarithmic base was optimized by gradient descent. In MATLAB, the repeated measures ANOVA tests are performed in two steps. First, a linear mixed effects model is fit to predict outcome values (in this case connectivity values) as a function of the repeated factors time (frames 1 to 8), stimulus type (word and PW) and the interaction between the two of them. Next, the resulting corrected beta-weighted values are grouped according to the factors time, stimulus, and the interaction of time and stimulus type, to characterize statistical differences using regular ANOVA tests (See Figure 4 for a visualization of the distribution of the marginal means of the data per functional network pairs included in this study). Mauchly’s test of sphericity was used to test for differences in variance among the groups being compared, and *p*-values were adjusted accordingly using the correction factor epsilon. Epsilon-adjusted *p*-values were corrected for multiple comparisons using the Tukey–Kramer method, and 0.05 was used as threshold for significance (Rudolph et al., 2018; Kovacs-Balint et al., 2019; Miranda-Domínguez et al., 2020).

### Methods’ recap

We aimed to characterize dynamic changes in functional connectivity in a task fMRI study where participants were asked to identify whether they were exposed to a word or a PW. Stimuli (word of PW) was shown for 2.5 s, and fMRI data was collected every 2.5 s from the beginning of the exposure and for 20 s in total. Trials were repeated several times. Each participant included in the study was exposed to two types of stimuli and we used the same amounts of trials to calculate personalized connectivity maps *via* connectotyping at each time point of each trial. Next, we used series of repeated measures ANOVA tests on group level data to identify changes in functional connectivity for each possible functional network pairs among six networks of interest, the Cingulo-Parietal, Default, Dorsal Attention, Fronto-Parietal, Salience, and Ventral Attention networks from the Gordon parcelation (Gordon et al., 2014). The ANOVA examined differences in functional connectivity for the interaction of time (frames 1–8) and stimulus type, for each network pair. Data are available from the corresponding author upon reasonable request. Code is available in https://fconn-anova.readthedocs.io/en/latest/

## Results

### Changes in functional connectivity for the interaction of time and stimulus type

**FIGURE 5:**
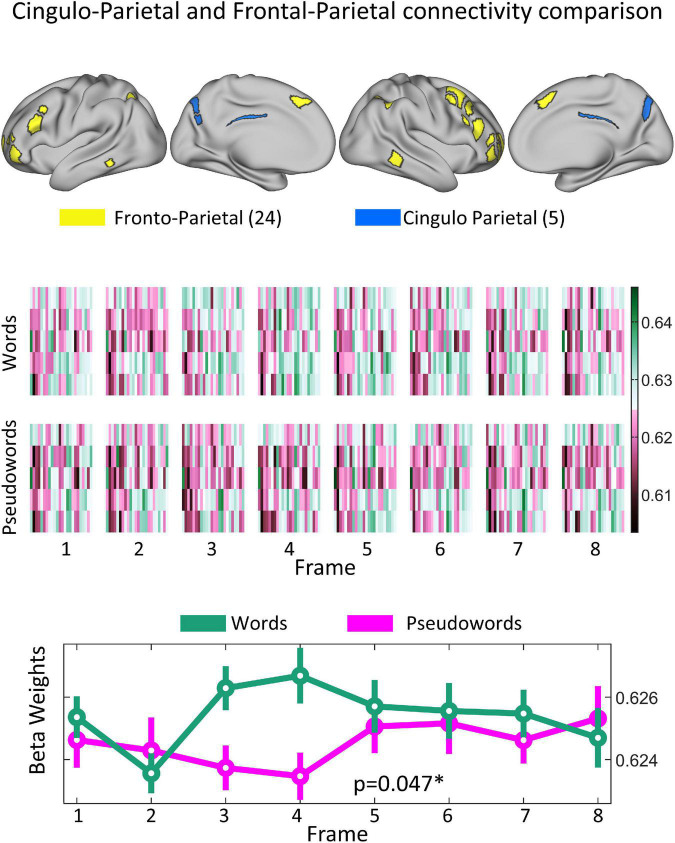
Significant difference observed in how the Cingulo–Parietal and Fronto–Parietal networks interact due to both progression of task and stimulus type. **(Top panel)** Shows the topological representation of cortical areas for both the Fronto-Parietal (yellow) and Cingulo Parietal (blue) networks. **(Middle panel)** Shows the mean sub-connectivity matrices between all the Cingulo Parietal and Fronto-Parietal ROIs (5 × 24 matrices) across participants for each stimuli (Word vs. Pseudowords) and each frame. The **(Bottom panel)** shows the distribution of marginal means of connectivity values for each frame and stimuli for connections belonging to the Fronto–Parietal and Cingulo–Parietal networks. Mean values shown as a circle and the dispersion is indicated with a bar covering 1.15 times the standard error of the connectivity values. When testing for how these values changed across frame and stimuli type, this functional system pair was found to be significant with a corrected *p*-value of 0.047.

The 8(time) × 2(condition) ANOVA showed a significant interaction between the Cingulo-Parietal and Fronto-Parietal Networks (*F* = 3.7155; *p* = 0.001, uncorrected; *p* = 0.047, corrected), as shown in Figure 5. *Post-hoc* analysis revealed that differences were driven by changes in connectivity values at frames 3 (paired *t*-test word vs non-word, *p* = 2.65e−5) and 4 (paired *t*-test word vs non-word, *p* = 2.68e−4). To note, we repeated this analysis including the Visual Network and found no significant results. The strongest effect was found for connectivity values between the Cingulo-Parietal and Fronto-Parietal networks (*p* = 0.06, corrected) but none of the functional network pairs including the Visual network showed differences for this interaction, as shown in Supplementary Figure 1.

### Robustness of results at different motion censoring thresholds

To test the robustness of our analysis using a more stringent threshold, we repeated analyses calculating connectotypes using a FD of 0.25. Only thirteen participants survived filtering at this threshold. While results did not pass corrections for multiple comparisons (*p* = 0.508, corrected) given the reduced sample size, the Cingulo-Parietal and Fronto-Parietal networks also exhibited the same temporal evolution in beta-weights for the interaction of time and stimulus type, as shown in Supplementary Figure 2A. In addition, we also recalculated our analysis with an FD of 0.5, allowing data from 17 participants, and found a similar difference in beta-weight response between these two networks. However, when correcting for multiple comparisons, the findings were not significant (*p* = 0.941, corrected) (Supplementary Figure 2B). These similarities in observed connectivity between the Cingulo-Parietal and Fronto-Parietal networks at different thresholds displayed a similar trend in temporal connectivity response as the original finding above.

### Characterizing changes in connectivity values in connectivity matrices calculated using pearson-correlations

**FIGURE 6:**
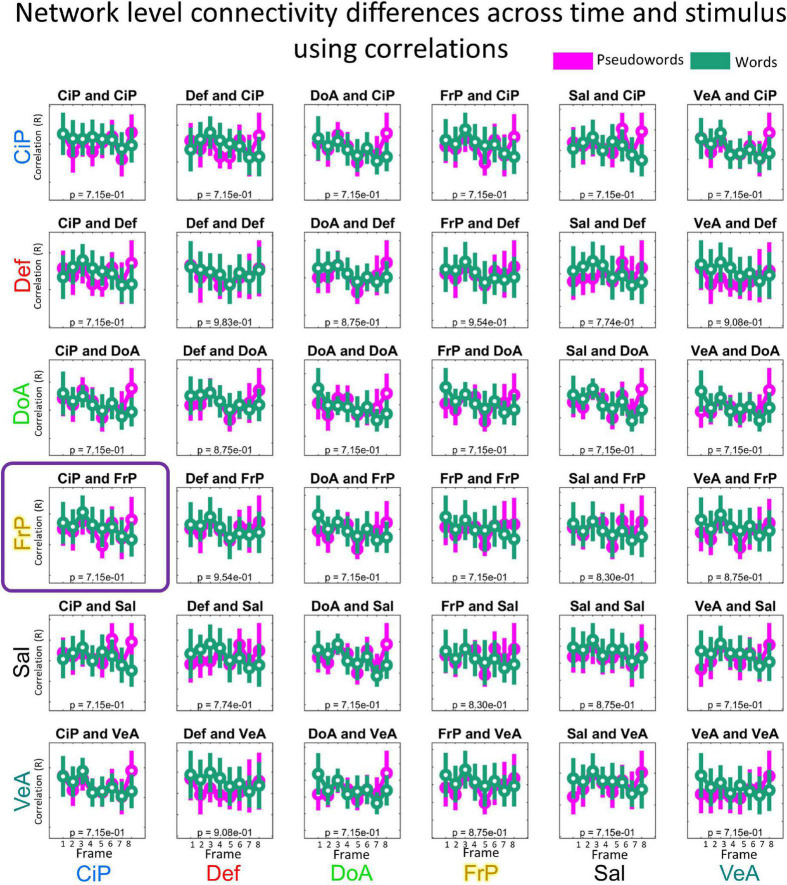
Distribution of mean connectivity values calculated using Pearson correlations per functional network pair and condition. Same description as in Figure 4.

We repeated all the previous analysis using connectivity matrices calculated *via* Pearson correlations instead of connectotypes using the same frames used to calculate connectotype. No FD threshold led to significant differences in functional connectivity. Figure 6 shows the distribution of marginal means of connectivity values when connectivity matrices were calculated using an FD threshold of 0.3 (i.e., the same threshold used for connectotyping). These data highlight potential improvements in fMRI analyses using connectotyping.

## Discussion

Recent advances in rs-fcMRI analysis approaches have led to increased understanding of brain functioning – where experimental designs have been able to identify brain areas supporting consciousness (Lloyd, 2002), moral judgment (Greene et al., 2001), as well as heritable patterns of brain connectivity (Miranda-Dominguez et al., 2018). Successful execution of mental tasks might require the collaboration of different brain networks in a timely manner. One approach that has been used to characterize dynamic changes in brain connectivity in task fMRI relies on correlations and averaging signals across participants (Hasson et al., 2004). For example, Najafi and colleagues used this approach to keep track of the changes in connectivity during anxious anticipation and found changes in connectivity between and within the Salience, Executive and Default Mode Networks (Najafi et al., 2017).

Given the noisy nature of functional MRI, some studies average data from multiple participants to improve the signal to noise ratio. While the result is a smoother signal, it comes at the price of blurring individual differences and dynamic changes in functional connectivity. In this study, we aimed to track temporal changes in brain connectivity during task performance using connectotyping, an efficient way to calculate functional connectivity between brain regions. Previously we showed that connectotyping can identify stable fingerprints efficiently (Miranda-Dominguez et al., 2014a,^2018^). Connectotyping models how the activity of each brain area can be modeled as the weighted contribution of all the other areas. Resulting beta-weights correspond to a functional connectivity matrix. Changes in functional connectivity at a particular functional network pair for the interaction of time and stimulus might indicate that that specific functional network responds differently to a given stimulus. Here we tested the viability of connectotyping on task data from a lexical decision-based fMRI study that used a widely spaced event-related design (∼20 s trials). The use of this particular dataset, capitalizing on the widely spaced design, allowed for the hemodynamic response function corresponding to a single stimulus to be detected without signal interference from the next or preceding stimulus. Our approach has the potential to reveal how functional connections between ROIs (i.e., here, at the network level) progress during the performance of a task not just at the peak of activation. As hypothesized, application of connectotyping to the word vs PW dataset revealed significant dynamic (i.e., across frames) connectivity differences between the Cingulo-Parietal and Fronto-Parietal networks, as a function of stimulus type (i.e., word vs. PW). Our interpretation of these findings is further elaborated below.

The significantly different dynamic temporal relation occurring as a function of stimulus type between the Cingulo-Parietal and Frontal-Parietal networks suggest that the evolving contributions between the Fronto-Parietal and the Cingulo-Parietal network are distinct in pattern depending on whether participants were viewing something meaningful (i.e., word) vs. meaningless (i.e., PW). It is important to mention that the proposed approach is able to discriminate between areas that respond specifically to the task because (a) the experimental design includes a contrast (Word vs PW) and (b) the repeated measures ANOVA is looking for differences in connectivity for the interaction of time and stimulus type. While there are other functional networks that also display dynamic changes, they are not distinct across stimuli (see for example Default and Cingulo-Parietal networks on Figure 4), hence they are not related to this task. Importantly, repeating analysis using connectivity matrices calculated *via* Pearson’s correlations, as opposed to connectotyping, did not have the resolving power to identify dynamic changes in brain connectivity.

After further testing and creating connectotypes with both more and less stringent movement thresholds (at 0.25 and 0.5 frame displacement thresholds), this observed Cingulo-Parietal and Frontal-Parietal network pattern of differentiated coactivation persisted, implying the stability of the findings (Supplementary Figure 2). Although these additional analyses did not withstand multiple-comparisons correction given the reduced sample size or signal to noise ratio, respectively, the presence of the same pattern of results supports the robustness of our primary finding.

The presence of dynamic connectivity differences between the Cingulo-Parietal and Fronto-Parietal networks support our principal hypothesis that task dependent regional brain communication changes during task progression; a finding that to our knowledge is the first of its kind. Our findings consequently also validate the use of connectotyping as a tool for task fMRI analysis which can provide a novel depiction of brain activity including dynamic temporal changes in functional connectivity. Additionally, we believe our findings are not simply a result of coactivation of networks. The original work by Nardos (2015) on this same dataset reported activation maps for the same contrast (Nardos, 2015). While there is some overlap for the Cingulo-Parietal network, most of the results. Nardos found that, in addition to areas within the Cingulo-Parietal network, areas belonging to the default, motor, ventral attention and Cingulo-Opercular system are behind the discrimination between words and PW (Nardos, 2015). In contrast, we found that dynamic changes in connectivity between the Cingulo-Parietal and Fronto-Parietal networks support discrimination between words and PW. Importantly, the Fronto-Parietal network was not identified as significant by Nardos. Since our findings do not coincide with the activation map, we do not believe that our analyses are a reflection of the activation of these networks.

Although our current approach differs from prior traditional task fMRI analyses, we did expect some overlap with findings from similar studies. Exposure to words vs. PWs resulted in significantly different temporal connectivity patterns between areas known to have a role in cognitive control, semantic processing, and memory retrieval. The Fronto-Parietal network is characterized as a task control network that has a particular role in the adaptive moment-to-moment requirements of a cognitive task such as task instantiation and dynamic feedback or error detection (Dosenbach et al., 2007). Cole et al. published evidence suggesting that the Fronto-Parietal network works as a cognitive hub by communicating with other control and processing networks to allow cognitive adaptation during tasks (Cole et al., 2013). This network also initiates and adjusts cognitive control to produce higher-level cognitive functions (Marek and Dosenbach, 2018). Here, the fact that such an adaptive control network displays distinct relations as a function of stimulus type is consistent with an expectation that resolution of the identity of a word vs. non-word may have different cognitive control demands.

The regions corresponding to the Cingulo-Parietal network have previously been linked with memory retrieval processes (Power et al., 2011). Parts of the Cingulo-Parietal network are found in the precuneus and near the posterior cingulate, regions that have previously been linked with semantic processing. For instance, the regions have been shown to distinguish between words and PWs in prior work using traditional fMRI analysis (Binder et al., 2009). The same two regions have also previously been associated with supporting word learning in young adults (Nardos, 2015). In addition, there is ample prior work that has associated those same two regions with memory retrieval (Yonelinas and Levy, 2002; Wheeler and Buckner, 2003; Dobbins and Wagner, 2005; Yonelinas et al., 2005; Cabeza et al., 2008; Nelson et al., 2010; Rugg and Vilberg, 2013). In aggregate, the aforementioned findings linking regions in the Cingulo-Parietal network with semantic processing and memory retrieval is consistent with our finding that dynamic functional connectivity between this network and the Fronto-Parietal network supporting adaptive cognitive control is what distinguishes meaningful words from meaningless PWs.

The proposed approach is unique in the fact that it tracks dynamic changes in whole brain functional connectivity contrasting the response to different stimuli at each functional network pair. This method does not require *a priori* knowledge of potential brain areas (seeds) involved in the task. This is made possible because we characterized functional connectivity using connectotyping (Miranda-Dominguez et al., 2014a,^2018^), a method with an improved signal-to-noise ratio (compared to traditional correlations) to characterize personalized maps of functional connectivity. In addition, the experimental design includes a contrast (word versus PW) enabling the identification of networks that respond differentially to each stimuli type. This contrast, we believe, makes possible the specificity to identify connections as opposed to merely co-activation. In other approaches, such as PPI, the distinction between co-activation and connectivity is made possible by including a regressor that is the product of the hypothesized hemodynamic response of the task and the timeseries of a seed that is *a priori* known to be involved in the task. It is important to mention that the proposed method is similar to Rissman connectivity (Rissman et al., 2004) in the fact that it aligns data according to their temporal evolution. In Rissman connectivity, the aligned data is used to estimate beta-weights associated with each event for every voxel. Resulting beta-weights are correlated across voxels to identify connections that respond to a given stimulus. Our approach, however, is different since we align whole-brain connectivity matrices and then characterize differences at each network pair for the interaction of time and stimulus type.

### Limitations and future work

Because of our stringent motion censoring, our analyses are based on the data of only 15 participants of a narrow age range, which may limit the generalizability of our results. In this exploratory study, due to our limited data and our focus on higher order heteromodal networks, we decided to exclude the primary sensory cortex in our analysis. Studies with a larger number of participants and different tasks might allow the inclusion of more networks and display additional significant interactions among networks. Our additional analysis including the visual network, however, supports our assumption that the visual network might not be involved in this particular paradigm of word discrimination. The usage of a widely spaced dataset was ideal to test the feasibility of using connectotyping to track dynamic changes in functional connectivity. A widely spaced design, however, limits the number of contrasts that can be performed and measured and might lead to fatigue in the participants. Fortunately, participants succeeded in identifying words and PW with an accuracy of 98% suggesting that this slow paradigm did not lead to reduced attention in the participants that could compromise our findings. As we succeed in using a linear model to track dynamic changes, superposition and convolution can be used in event-related experiments where stimuli can be changed at each TR. By applying those validated methods to deconvolve the beta-weights corresponding to each frame and stimulus, the same statistical analysis (i.e., repeated measures ANOVA) can be used to track dynamic changes in functional connectivity. This approach is something we intend to continue exploring using task data from the Adolescent Brain Cognitive Development (ABCD) Study (Casey et al., 2018).

## Conclusion

Task execution requires the orchestrated involvement of different brain networks. Here we showed that by calculating connectivity matrices using connectotyping at each time point during the execution of a task, we can identify the changes in brain connectivity that support semantic discrimination in a word versus PW paradigm using fMRI. We showed that connectotyping has a resolving power that cannot be achieved by using traditional correlations. While limited by the constraints of our data and the novelty of our approach, our group level findings serve to expand on the roles and functions of the Cingulo-Parietal and Fronto-Parietal networks as an incentive for others to pursue analyses which account for patterns of dynamic whole-brain connectivity and provide temporal resolution. The application of connectotyping to additional studies exploring other tasks and with differentially spaced study designs will not only further validate the use of this approach but also has the potential to expand our understanding of brain activity during the performance of a task.

## Data availability statement

The original contributions presented in this study are included in the article/Supplementary material, further inquiries can be directed to the corresponding author.

## Ethics statement

The studies involving human participants were reviewed and approved by the Washington University Human Studies Committee (IRB ID # 201202083). All patients/participants provided their written informed consent to participate in this study.

## Author contributions

VV-T, BN, BS, DF, and OM-D contributed to the conception and design of the study. BN performed the data collection. VV-T and BN processed the neuroimaging data. VV-T, BN, and OM-D performed the statistical analysis and wrote the first draft of the manuscript. VV-T, BS, DF, and OM-D read and approved the submitted version. All authors contributed to the manuscript revision.

## Footnotes

1
https://github.com/DCAN-Labs

## Supplementary material

Supplementary Figure 1 Distribution of mean connectivity values per functional network pair and condition including the visual network. Beta-weights were calculated for each condition as indicated in our experimental design (Figure 3) and grouped by functional network pair. Each boxplot highlights the mean values using a circle and the dispersion is indicated with a bar covering 1.15 times the standard deviation of the connectivity values. Data is color-coded by stimuli: pseudoword (purple) and word (green). *X*-axis indicates the time, in frames (TR of 2.5 s each). In this study we included the following networks: Cingulo-Parietal (CiP, *n* = 5 Regions of Interest), Default (Def, *n* = 41), Dorsal Attention (DoA, *n* = 32), Fronto-Parietal (FrP, *n* = 24), Salience (Sal, *n* = 4), and Ventral Attention (VeA, *n* = 23) Visual (Vis, *n* = 39).

Supplementary Figure 2 Difference of how the Cingulo-Parietal and Fronto-Parietal networks interact over time at other thresholds of head-movement suggest robust initial finding. The **(A)** shows the change in beta-weights between the Fronto-Parietal and Cingulo-Parietal networks at a movement threshold of 0.25 mm. After correcting for multiple comparisons, this functional system pair was not found to be significant (*p* = 0.508). The **(B)** shows the results from the same analysis as the left information when the movement threshold was set at a higher value of 0.5mm. When this data underwent corrections for multiple comparisons, this functional system pair was not found to be significant (*p* = 0.941).
