# Supplementary figures and images for: Use of connectotyping on task functional MRI data reveals dynamic network level cross talking during task performance
Source: Front Neurosci. 2022 Oct 10;16:951907. doi: 10.3389/fnins.2022.951907 (PMC9589037; doi:10.3389/fnins.2022.951907)

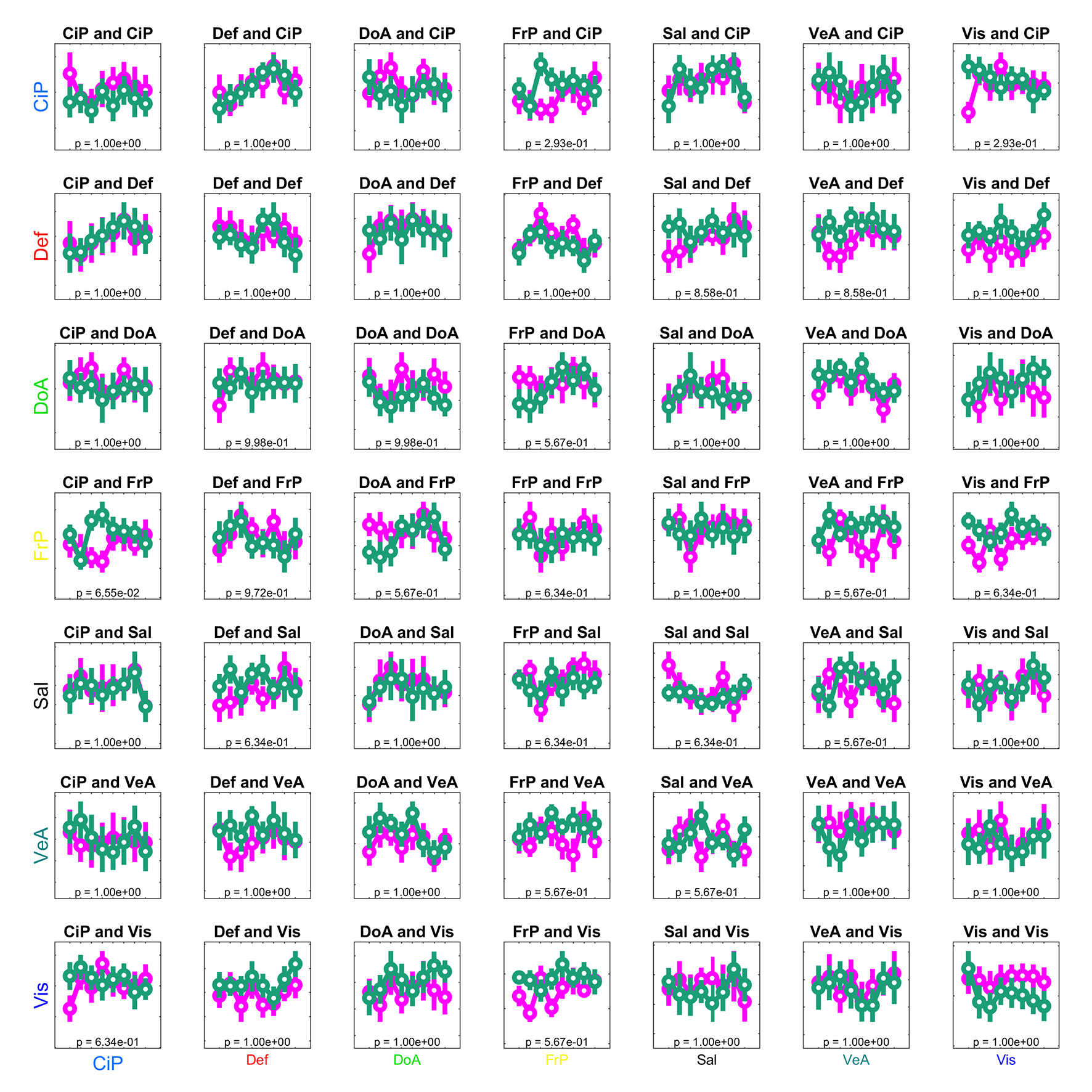

Supplement: Supplementary Figure 1 — Distribution of mean connectivity values per functional network pair and condition including the visual network. Beta-weights were calculated for each condition as indicated in our experimental design (Figure 3) and grouped by functional network pair. Each boxplot highlights the mean values using a circle and the dispersion is indicated with a bar covering 1.15 times the standard deviation of the connectivity values. Data is color-coded by stimuli: pseudoword (purple) and word (green). X-axis indicates the time, in frames (TR of 2.5 s each). In this study we included the following networks: Cingulo-Parietal (CiP, n = 5 Regions of Interest), Default (Def, n = 41), Dorsal Attention (DoA, n = 32), Fronto-Parietal (FrP, n = 24), Salience (Sal, n = 4), and Ventral Attention (VeA, n = 23) Visual (Vis, n = 39). [file Image_1.TIF]

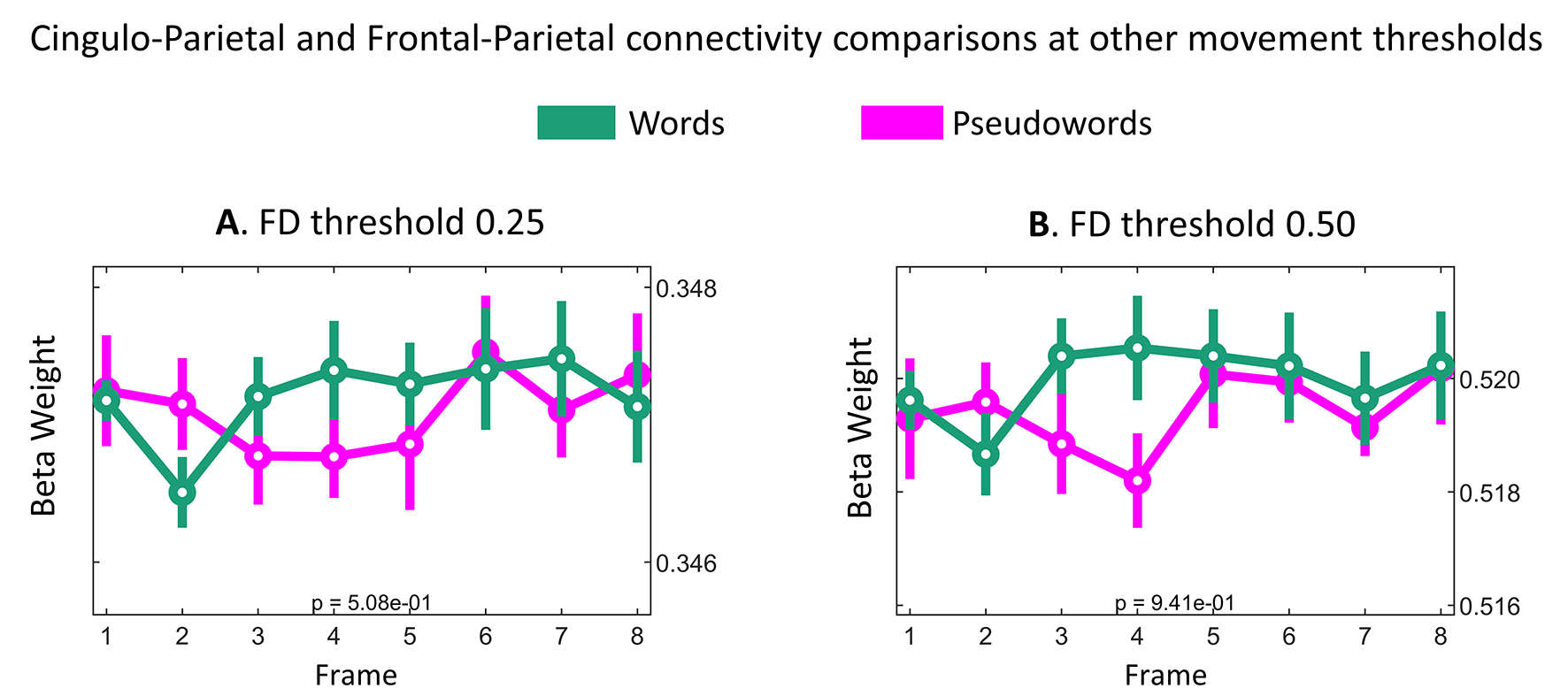

Supplement: Supplementary Figure 2 — Difference of how the Cingulo-Parietal and Fronto-Parietal networks interact over time at other thresholds of head-movement suggest robust initial finding. The (A) shows the change in beta-weights between the Fronto-Parietal and Cingulo-Parietal networks at a movement threshold of 0.25 mm. After correcting for multiple comparisons, this functional system pair was not found to be significant (p = 0.508). The (B) shows the results from the same analysis as the left information when the movement threshold was set at a higher value of 0.5mm. When this data underwent corrections for multiple comparisons, this functional system pair was not found to be significant (p = 0.941). [file Image_2.TIF]
